# Epidemiological characteristics for patients with traumatic brain injury and the nomogram model for poor prognosis: an 18-year hospital-based study

**DOI:** 10.3389/fneur.2023.1138217

**Published:** 2023-05-23

**Authors:** Shaochun Guo, Ruili Han, Fan Chen, Peigang Ji, Jinghui Liu, Yulong Zhai, Min Chao, Wenjian Zhao, Yang Jiao, Chao Fan, Tao Huang, Na Wang, Shunnan Ge, Yan Qu, Yuan Wang, Liang Wang

**Affiliations:** ^1^Department of Neurosurgery, Tangdu Hospital, The Fourth Military Medical University, Xi'an, China; ^2^Department of Neurosurgery, The Second Affiliated Hospital of Shaanxi University of Chinese Medicine, Xi'an, China; ^3^Department of Anesthesiology, Tangdu Hospital, The Fourth Military Medical University, Xi'an, China; ^4^Innovation Center for Advanced Medicine, Tangdu Hospital, The Fourth Military Medical University, Xi'an, China

**Keywords:** epidemiology, traumatic brain injury, clinical characteristics, mortality, prognostic nomogram

## Abstract

**Objective:**

Traumatic brain injury (TBI) is a global social, economic, and health challenge that is associated with premature death and long-term disability. In the context of rapid development of urbanization, the analysis of TBI rate and mortality trend could provide abundant diagnosis and treatment suggestions, which helps to form future reference on public health strategies.

**Methods:**

In this study, as one of major neurosurgical centers in China, we focused on the regime shift of TBI based on 18-year consecutive clinical data and evaluated the epidemiological features. In our current study, a total of 11,068 TBI patients were reviewed.

**Results:**

The major cause of TBI was road traffic injuries (44.%), while the main type of injury was cerebral contusion (*n* = 4,974 [44.94%]). Regarding to temporal changes, a decreasing trend in TBI incidence for patients under 44 years old was observed, while an increasing trend for those aged over 45 years was indicated. Incidences of RTI and assaults decreased, while ground level fall presented increasing incidences. The total number of deaths was 933 (8.43%), with a decreasing trend in overall mortality since 2011. Age, cause of injury, GCS at admission, Injury Severity Score, shock state at admission, trauma-related diagnoses and treatments were significantly associated with mortality. A predictive nomogram model for poor prognosis was developed based on patient's GOS scores at discharge.

**Conclusions:**

The trends and characteristics of TBI patients changed with rapid development of urbanization in the past 18 years. Further larger studies are warranted to verify its clinical suggestions.

## Introduction

Globally, more than 50 million people suffer from traumatic brain injury (TBI) each year (1, 2), and it is the leading cause of death and disability for all ages (3). Annually, TBI is estimated to cost the global economy approximately US$400 billion. There is an urgent need to focus on the prevention, treatment, and research of TBI to reduce the burden and social cost (4, 5).

As a developing economical entity, China has experienced rapid urbanization in the past decades, which has had great influences on every aspect of social-economical activities, especially the medical system. Data from several large population-based studies conducted in the 1980's showed that TBI incidences in China were much lower than that in high-income countries, reflecting the incomplete demographics of Chinese patients with TBI (6). Over time, changes have occurred in the TBI landscape in China (5–9). In terms of treatment methods, a national emergency and critical care campaign has been started since the outbreak of SARS in 2003, together with the advancement of legislation, optimized emergency treatment policies, and clinical management (5–10). In 2020, the basic characteristics of TBI and the level of trauma treatment in China have been reported, indicating great advancements in the past decades (9). However, considering the imbalanced development of China, elucidation of the characteristics of different regions and exploration of the regularities behind urbanization is still needed to guide future TBI treatment, especially in developing countries.

The essence of emergency neurotrauma care is rapid assessment, decision-making, and treatment. It has been limited due to a lack of objective standard guidelines and validated risk factor models for poor prognosis to predict the outcomes of interest (11). The establishment of predictive models for patients with TBI could enhance the speed of the decision-making process for patients, families, and healthcare professionals with objective treatments (12).

In our current study, we retrospectively retrieved data over the past 18 years from 11,068 patients with TBI in a tertiary neurosurgical center in western China and analyzed the demographic characteristics, trends, death, and prognosis. A prediction nomogram model for poor prognosis was developed based on Glasgow Outcome Scale (GOS) scores for patients discharged from the hospital, which may help guide future treatment and decision-making for patients with TBI.

## Materials and methods

### Study design and patient selection

In this study, 11,068 patients with TBI who were treated at the Neurosurgery Trauma Center, Tangdu Hospital, Xi'an, from 1 January 2003 to 31 December 2020, were retrieved for retrospective assessment. The inclusion criteria were as follows: i. patients with TBI with confirmed brain injury, either at emergency department (ED) examination or other hospital examination within 24 h after brain injury and ii. patients with definite brain injury, multiple injuries, or decreased consciousness.

The exclusion criteria were as follows: i. penetrating brain injury and related spinal cord injury; ii. lactating and pregnant women; iii. patients with other malignant tumors, severe mental disorders, or hematological diseases; iv. patients who died in the emergency department; and v. patients with a history of neurosurgery, which may affect the judgment of the brain trauma condition.

This study adhered to the principles of the Declaration of Helsinki. All medical records were anonymized, and no patient information was extracted except for research purposes. The Institutional Research Board of Tangdu Hospital, The Fourth Military Medical University, approved this study (TDLL-202207-08).

### Data source

All eligible patients were reviewed by two researchers. The demographic variables, causes of injury, clinical features, and prognosis (including death) data at admission were recorded for subsequent analysis.

Based on age, patients were divided into the following 4 groups: i. 0–14 years; ii. 15–44 years; iii. 45–64 years; and iv. over 65 years. The number of patients among groups was compared. According to the Glasgow Coma Scale (GCS), TBI severity was divided into the following three levels: i. mild—GCS scores of 13 to 15; ii. moderate—GCS scores of 9 to 12; and iii. severe—GCS scores of 3 to 8 (13). Extracranial trauma injury was quantified by the injury severity score (ISS) as follows: ISS scores of 1 to 8 denoted mild to moderate injury, ISS scores of 9 to 15 denoted serious injury, ISS scores of 16 to 24 denoted severe injury, and ISS scores of 25 to 75 denoted critical injury (14).

The causes of injury included road traffic injuries (RTI), falls from heights (FFH), ground-level falls (GLF), assaults, and others (violence and attempted suicide). RTI refers to personal injuries as a result of the motor vehicle and non-motor vehicle accidents in squares, public parking lots, and other places that are used for public passage. FFH refers to brain injuries due to falling from a high place (above the ground) and being impacted by high speed in daily work or life. GLF refers to head injuries due to accidentally falling on the ground. Assault refers to injuries that arise as a result of hitting a limb with a fist, foot, or instrument.

### Definition of clinical diagnosis and treatment

Clinical diagnoses were made according to the patient's state of consciousness, vital signs, and CT scan findings. These diagnoses included cerebral contusion (CC), traumatic subarachnoid hemorrhage (T-SAH), acute subdural hematoma (A-SDH), skull fractures (including the base of skull fractures, SF), acute epidural hematoma (A-EDH), diffuse axial cord injury (DAI), and others (scalp trauma and intracranial infection). The concomitant diagnosis refers to TBI accompanied by injury to other parts of the body, including chest injury, limb injury, and others (abdominal injury and pelvic fractures).

Based on medical interventions, patients were divided into non-surgical and surgical groups. The surgical group was further divided into the following three subgroups based on surgical manipulation type: intracranial pressure (ICP) sensor insertion, craniotomy (CR), decompression craniectomy (DC), and others [external ventricular drainage (EVD) and reduction of skull fracture].

The GOS prognosis scores for all patients were determined at discharge and defined as follows: GOS scores of 1–3 denoted poor prognosis (including death), while GOS scores of 4–5 denoted good prognosis.

### The nomogram prediction model

We established a nomogram prediction model for poor prognosis. Using “rms” in R, univariate and multivariate logistic regression analyses with stepwise backward were performed to screen the risk factors associated with poor prognosis. To obtain comparable odds ratios (ORs) for linear relationships and to attain clear threshold values for continuous variables, each variable was rescaled using the receiver operating characteristic (ROC) curve. Logistic regression models were established by including the risk factors, with poor prognosis as the prediction. Based on existing data, patients were randomly assigned to training and experimental groups. Patients (89.89%, *n* = 9,949) from 2003 to 2017 were assigned to the training cohort, while patients (10.11%, *n* =1,119) from 2018 to 2020 were taken as the validation cohort. There was no significant difference between the two cohorts (Supplementary Table 1). Optimal model selection was performed by applying a backward stepwise selection procedure. A nomogram was constructed based on risk factors from the multivariate logistic regression test. The nomogram's prediction accuracy was evaluated by calibration curve analyses. Finally, the model was externally validated in a separate cohort.

### Statistical analyses

Descriptive statistics were used to characterize categorical and numerical variables. Categorical data were tabulated and presented as number(s) and percentage(s). Non-parametric/continuous variables (age, total length of stay, GCS, and ISS) were presented as medians and interquartile ranges (IQR), while categorical variables such as sex, causes of injury, type of injury, and survival status were presented as numbers and percentages. Univariate (chi-square test, *t*-test, or Mann-Whitney U test, as appropriate) and multivariate logistic regression analyses were used to analyze clinical variables associated with death to identify risk factors. Analyses were performed using IBM Statistical Package for Social Sciences (SPSS), version 23, and RStudio (1.0.136), and a *p*-value of ≤ 0.05 was the threshold for statistical significance.

## Results

**Figure 1:**
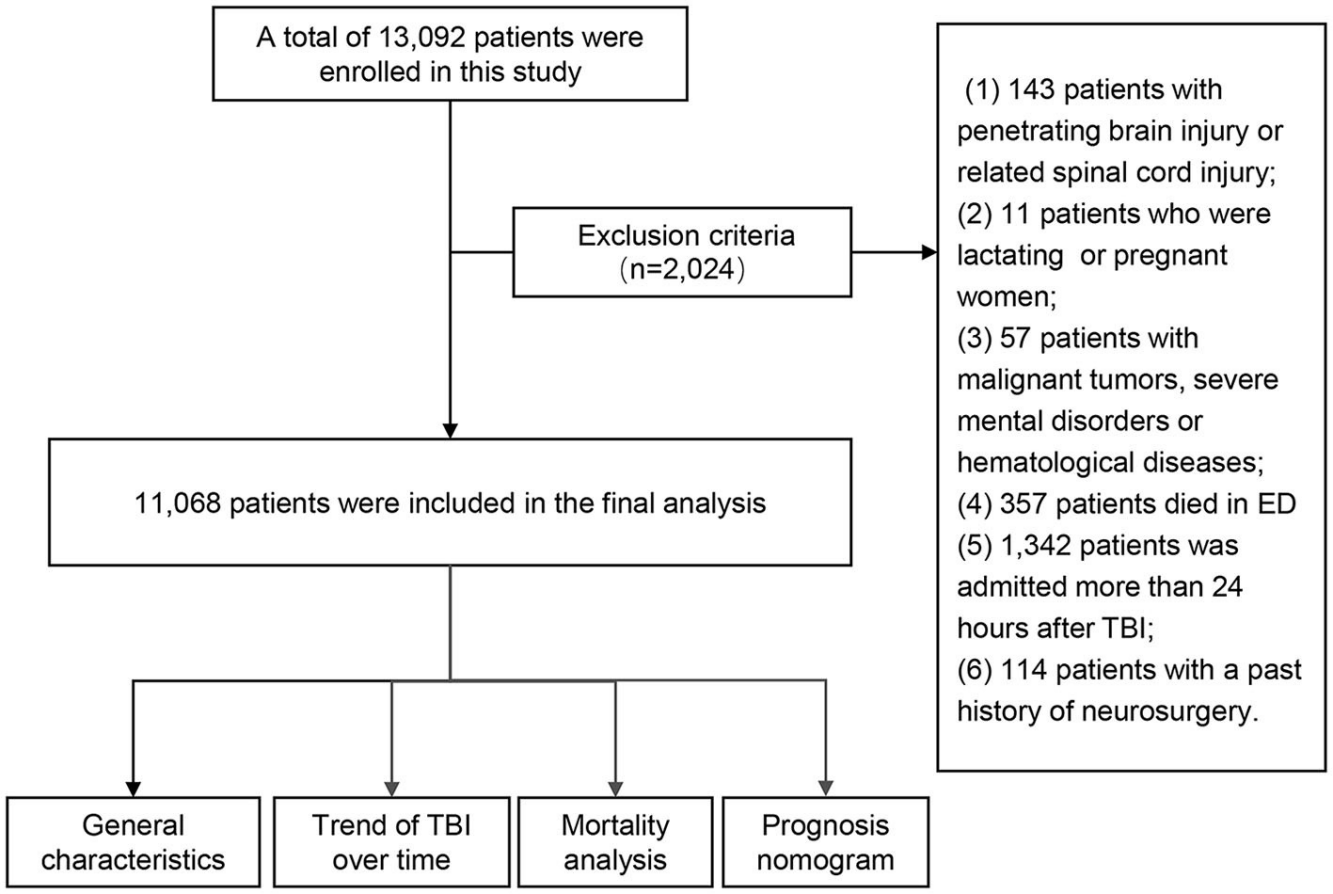
Diagram of the study procedure. TBI, traumatic brain injury; ED, emergency department.

A total of 13,092 patients were enrolled in this study. Due to insufficient parameters for certain cases, 2,024 patients were excluded. Finally, 11,068 patients were included in the final analysis (Figure 1).

### Demographic and general characteristics

**Figure 2:**
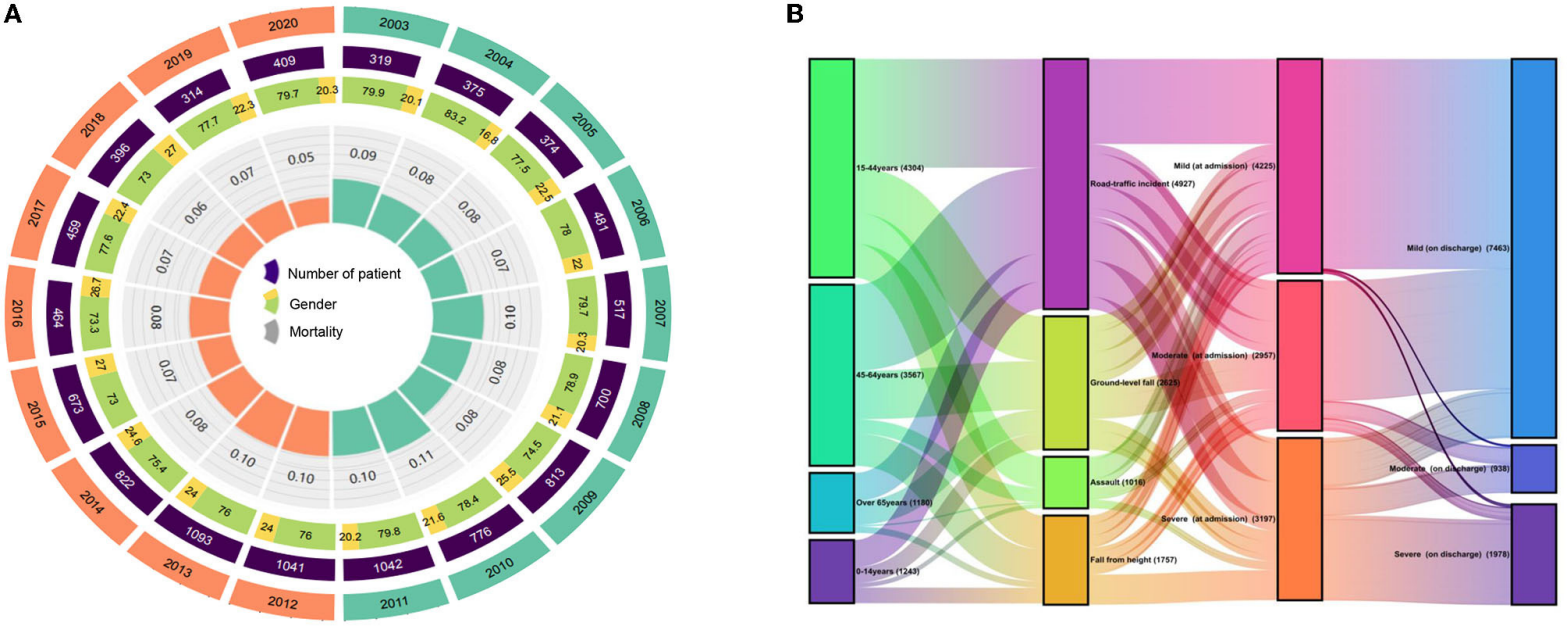
**(A)** Demographic and general characteristics of the patients. **(B)** Changes between age, cause of injury, severity at admission, and severity at discharge in patients with TBI.

Most of the patients were male (77.06%; *n* = 8,529), with a median age of 43 years (IQR 25–56). Common causes of TBI were RTI, FFH, and GLF (44.46, 23.73, and 15.64%, respectively). The median GCS score at admission was 14 (IQR 12–15), of which 3,412 (30.83%) patients presented with severe TBI (GCS 3–8). The median ISS score at admission was 15 (IQR 9–24), with 1,624 (14.67%) patients presenting critical trauma (ISS 25–75). The most common diagnosis was CC (44.94%, *n* = 4,974), followed by T-SAH (42.56%, *n* = 4,711) and A-SDH (28.22%; *n* = 3,123). Concomitant diagnosis with TBI was frequently observed (50.75%, *n* = 5,617), with chest injuries being the most common (12.31%, *n* = 1,362). A total of 5,641 (50.97%) patients were treated with surgical interventions, with CR (28.84%, *n* = 1,627) being the most common surgical approach. The demographic and general characteristics of the patients are summarized in Table 1 and Figure 2A. The age group, causes of injury, severity at admission, and severity at the discharge of patients with TBI are illustrated in Figure 2B. The onset time of patients with TBI showed certain regularity in the month, week, and day (Supplementary Figure 1).

**Table 1:** Demographic and general characteristics of the patients in the study.

**Demographic characteristics**		**Overall (*n* = 11,068)**
Sex	Male	8,529 (77.06%)
	Female	2,539 (22.94%)
Age (years)	Median	43 (IQR 25–56)
	Mild	40 (IQR 22–55)
	Moderate	45 (IQR 25–56)
	Severe	46 (IQR 30–58)
	0–14 years	1,302 (11.76%)
	15–44 years	4,595 (41.52%)
	45–64 years	3,860 (34.88%)
	Over 64 years	1,311 (11.84%)
ICU length (d)	Median	2 (IQR 0–5)
Total length of stay (d)	Median	10 (IQR 5–16)
	Mild	9 (IQR 6–14)
	Moderate	11 (IQR 7–18)
	Severe	10 (IQR 3–19)
	≤1 d	776 (7.01%)
	1–7 d	2,967 (26.79%)
	7–14 d	3,898 (35.19%)
	14–30 d	2,610 (23.56%)
	>30 d	825 (7.45%)
**Clinical presentation**		
Cause of injury	Road traffic injuries	4,921 (44.46%)
	Falls from heights	1,731 (15.64%)
	Falls from the ground	2,626 (23.73%)
	Assaults	1,016 (9.18%)
	Other	774 (6.99%)
GCS score at admission	Median	14 (IQR 12–15)
	Mild (13–15)	4,506 (40.71%)
	Moderate (9–12)	3,150 (28.46%)
	Severe (3–8)	3,412 (30.83%)
ISS score at admission	Median	15 (IQR 9–24)
	Mild to moderate (1–8)	2,154 (19.46%)
	Serious (9–15)	3,836 (34.66%)
	Severe (16–25)	3,458 (31.24%)
	Critical (25–75)	1,624 (14.67%)
Diagnosis	CC	4,974 (44.94%)
	T-SAH	4,711 (42.56%)
	A-SDH	3,123 (28.22%)
	SF	2,324 (21.00%)
	A-EDH	2,087 (18.86%)
	DAI	353 (3.19%)
	Other	1,121 (12.15%)
Concomitant diagnosis	Total	5,617 (50.75%)
	Chest injuries	1,362 (12.31%)
	Limb injuries	1,178 (10.64%)
	Other	3,077 (27.80%)
Shock	Shock	1,014 (9.16%)
	Non-shock	10,054 (90.84%)
Treatment	Non-surgical	5,427 (49.03%)
	Surgical	5,641 (50.97%)
Surgical manipulation type	ICP	871 (15.44%)
	CR	1,627 (28.84%)
	DC	111 (1.97%)
	ICP + CR	287 (5.09%)
	ICP + DC	342 (6.06%)
	CR + DC	1,031 (18.28%)
	ICP + CR + DC	418 (7.41%)
	Other (EVD, Reduction of skull fracture)	954 (16.91%)

GCS, Glasgow Coma Scale; ISS, injury severity score; CC, cerebral contusion; T-SAH, traumatic subarachnoid hemorrhage; A-SDH, acute subdural hematoma; SF, skull fractures (including the base of skull fractures); A-EDH, acute epidural hematoma; DAI, diffuse axial cord injury; ICP, intracranial pressure sensor insertion; CR, craniotomy; DC, decompression craniectomy; EVD, external ventricular drainage.

### The TBI-related trend over 18 years

During 2003–2020, the total number of inpatients, gender, and mortality rates per year exhibited dynamic variations, while mortality rates slightly increased from 2003 to 2011 and thereafter significantly decreased (Figure 2A). The decrease in mortality rates might be attributed to changes in treatment modalities. For instance, the death rate has markedly reduced since the introduction of ICP sensor insertion in 2011.

**Figure 3:**
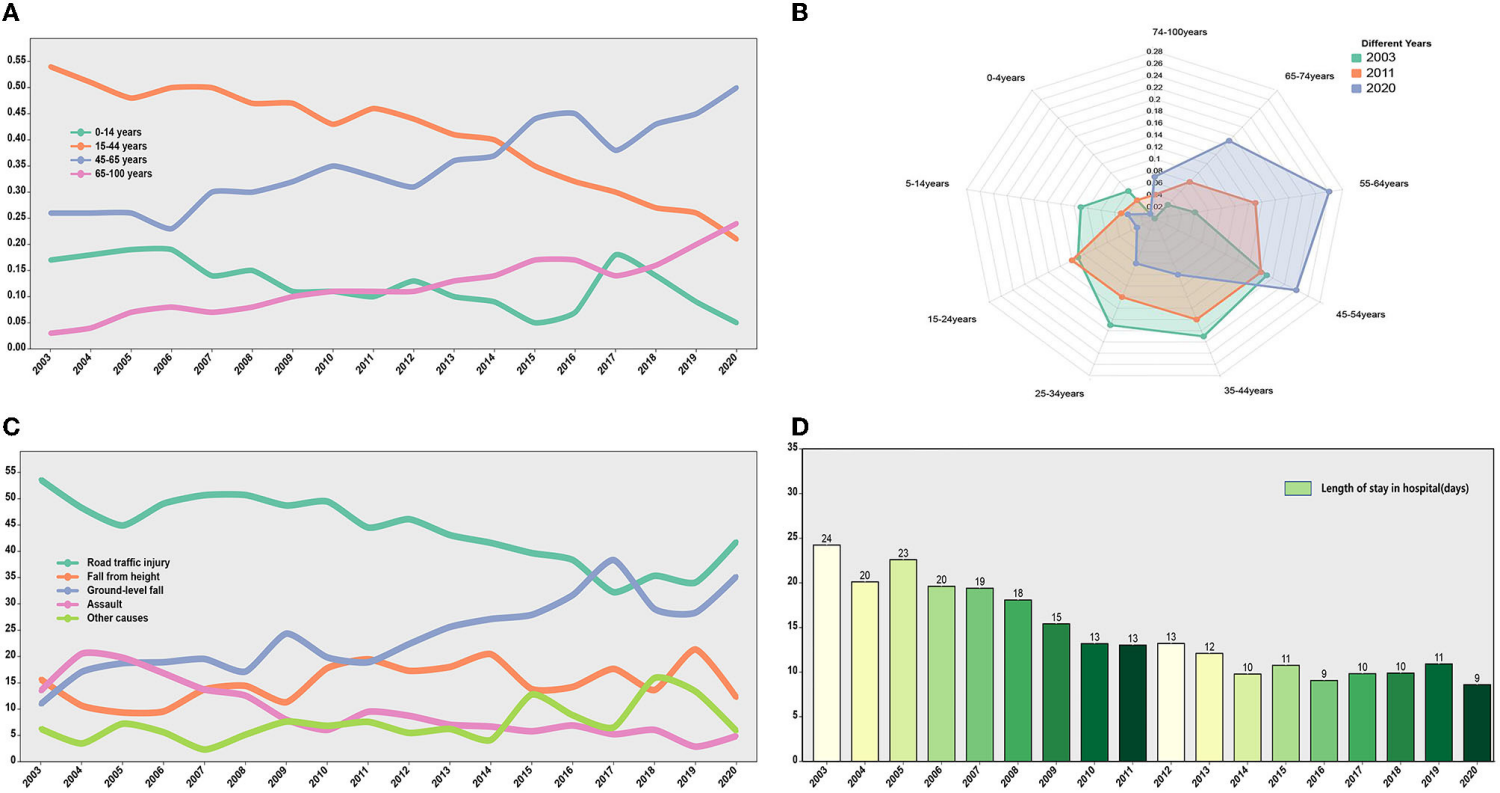
The TBI-related trend over 18 years. **(A)** The trend of TBI incidence rate over time in different age groups. The proportion of TBI incidence in age groups younger than 44 years showed a downward trend. The proportion of TBI incidence in age groups older than 44 years showed an upward trend. **(B)** The distribution of TBI age groups in different years (2003, 2011, and 2020). **(C)** The trend of injury mechanisms over time. **(D)** Length of stay in hospital (days) over time.

From 2003 to 2020, the general characteristics of patients with TBI showed a certain trend over time. The trend was more pronounced for age, cause of injury, and length of hospital stay. We found that the proportions of TBIs in two age groups below 44 years decreased and the proportions of TBIs in two age groups above 45 years increased, especially in patients over 65 years of age (Figure 3A). The distribution of patients with TBI in different age groups varied greatly in different years. Compared with 2003, the population with the disease in 2020 is getting older (Figure 3B). Among the causes of injury, the proportions of RTI and assaults tended to decrease (53.6 to 41.8% and 13.5 to 4.9%, respectively), while the proportions of GLF tended to increase (13.5 to 35.2%) (Figure 3C). Besides, the median length of stay for patients showed a downward trend, from 14 days in 2003 to 8 days in 2020 (Figure 3D). The changes in other characteristics of patients with TBI (gender, surgical method, severity of GCS based on admission, and severity of ISS based on admission) showed a certain trend over time (Supplementary Figure 2).

Furthermore, the first 9 years (2003–2011) and the second 9 years (2012–2020) were divided into two groups. All factors of the patients were analyzed and compared in the two time periods, and the differences in GCS, ISS, injury causes, treatment, and prognosis were found to be statistically significant (*p* < 0.05) (Table 2).

**Table 2:** Univariate and multivariate analyses between the first 9 years and the last 9 years.

		**Univariate analyses**		**Multivariate analyses**	
		***P*** **-value**	**OR (95% CI)**	***P*** **-value**	**OR (95% CI)**
GCS score at admission	Mild (13–15)	Ref	Ref	Ref	Ref
	Moderate (9–12)	0.001	0.769 (0.701–0.842)	0.001	0.681 (0.614–0.755)
	Severe (3–8)	0.001	1.2 (1.098–1.313)	0.667	1.024 (0.919–1.14)
ISS score at admission	Mild to moderate (1–8)	Ref	Ref	Ref	Ref
	Serious (9–15)	0.001	0.657 (0.591–0.731)	0.001	0.79 (0.704–0.887)
	Severe (16–25)	0.57	0.969 (0.87–1.08)	0.042	0.879 (0.777–0.995)
	Critical (over 25)	0.072	1.127 (0.99–1.283)	0.073	0.836 (0.688–1.017)
Cause of injury	Road traffic injuries	Ref	Ref	Ref	Ref
	Falls from heights	0.001	1.399 (1.254–1.562)	0.001	1.455 (1.289–1.641)
	Falls from the ground	0.001	1.759 (1.597–1.937)	0.001	1.781 (1.641–1.982)
	Assaults	0.001	0.653 (0.568–0.751)	0.001	0.703 (0.602–0.82)
	Other	0.001	1.536 (1.318–1.791)	0.001	1.492 (1.261–1.766)
Treatment	Non-surgical	Ref	Ref	Ref	Ref
	Surgical	0.001	2.206 (2.044–2.38)	0.001	1.786 (1.631–1.954)
Prognosis	Good prognosis	Ref	Ref	Ref	Ref
	Poor prognosis	0.001	0.836 (0.76–0.92)	0.001	1.388 (1.19–1.619)

### Mortality

**Figure 4:**
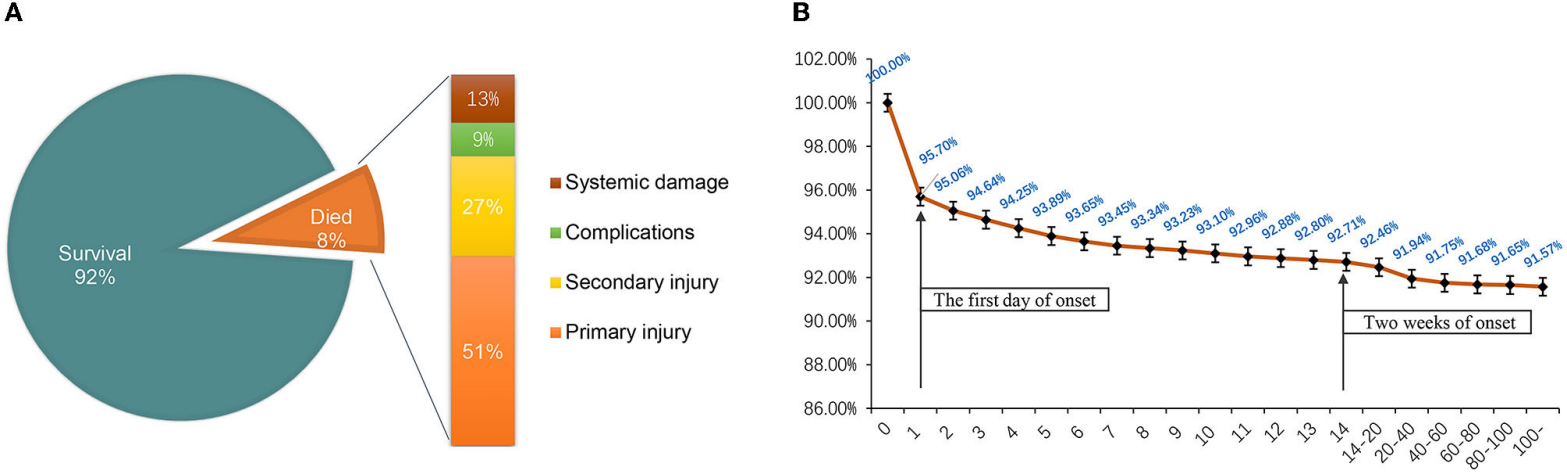
**(A)** Mortality ratio and causes of death of hospitalized patients. **(B)** Survival of hospitalized patients over time. The death peak was on the first day of onset, and most patients died within 2 weeks of hospitalization.

**Figure 5:**
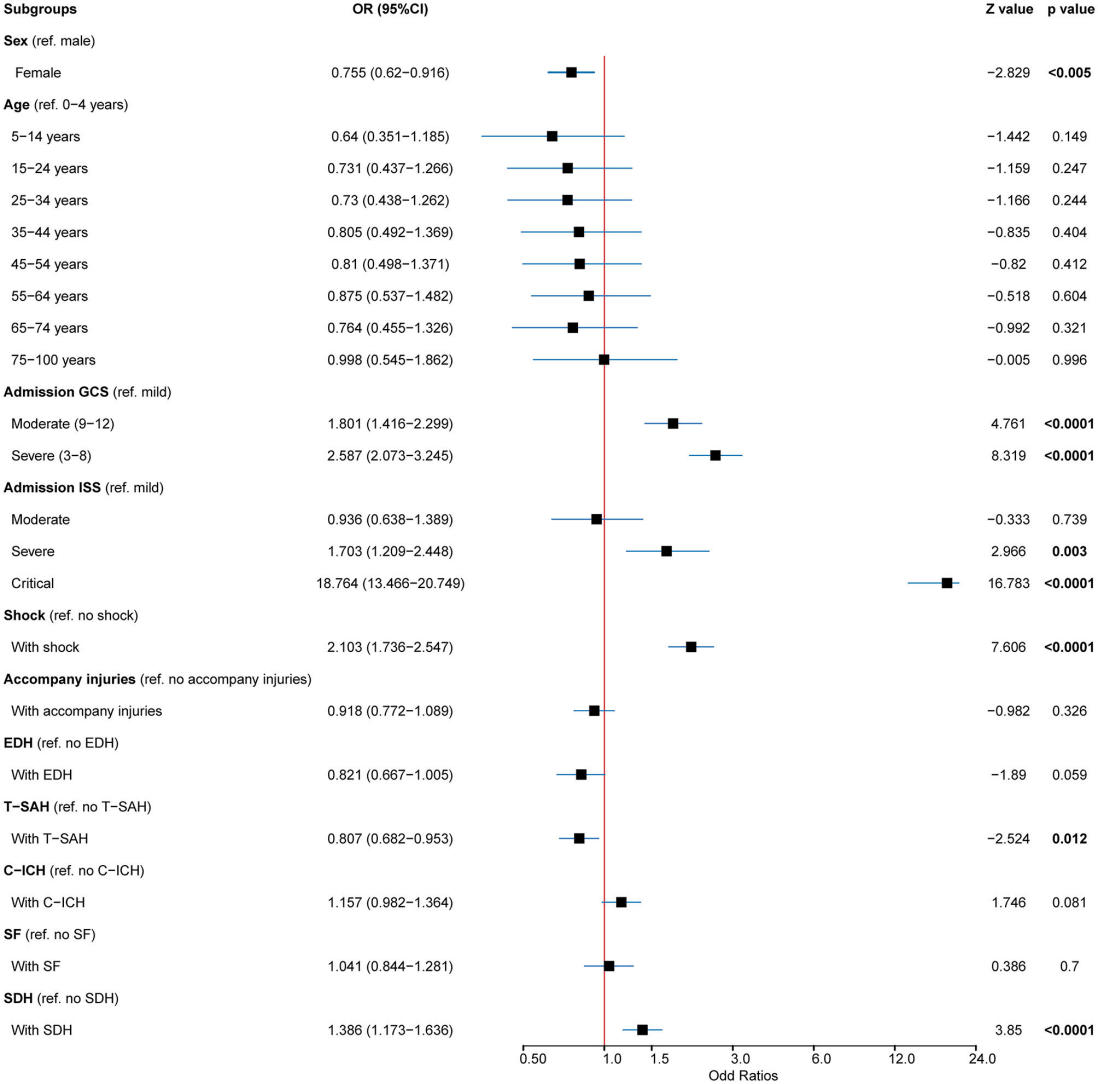
Predictors for mortality in patients with TBI. Multivariable analysis in patients with TBI showed that the mortality predictors for cases included age, GCS, ISS, shock, A-SDH, A-EDH, and T-SAH. TBI, traumatic brain injury; GSC, Glasgow Coma Scale; ISS, injury severity score; A-SDH, acute subdural hematoma (A-SDH); A-EDH, acute epidural hematoma; T-SAH, traumatic subarachnoid hemorrhage. Error bars represent 95% CI.

A total of 933 (8.43%) patients died during hospitalization; among them, 818 (23.97%) were patients with severe TBI. The causes of death were reviewed, with primary cerebral injury (51.02%, *n* = 476) as the dominant cause, followed by secondary injuries (26.69%, *n* = 249), such as cerebral edema and postoperative hypercranial pressure; complications (9.00%, *n* = 84), including intracranial infection and pulmonary infection; and systemic injury (13.29%, *n* = 124), including multiorgan functional deficit (Figure 4A). The survival of patients during hospitalization varied with the length of hospitalization, among which the death of patients on the first day of admission was the most common (Figure 4B). Univariate analysis showed that age, cause of injury, GCS at admission, ISS at admission, shock state at admission, TBI-related diagnoses (CC, T-SAH, A-SDH, SF, and A-EDH), and treatments were significantly associated with mortality (Table 3). Further binary logistic analysis revealed seven potential risk factors for inpatient mortality as follows: sex (female vs. male) (OR, 0.755; 95% CI, 0.62–0.916; *p* < 0.005), GCS (moderate vs. mild) (OR, 1.801; 95% CI, 1.416–2.299; *p* < 0.001), GCS (severe vs. mild) (OR, 2.587; 95% CI, 2.073–3.245; *p* < 0.001), ISS (severe vs. mild) (OR, 1.703; 95% CI, 1.209–2.448; *p* < 0.001), ISS (critical vs. mild) (OR, 18.764; 95% CI, 13.466–20.749; *p* < 0.001), shock (OR 2.103; 95% CI, 1.736–2.547; *p* < 0.001), A-SDH (OR 1.386; 95% CI, 1.173–1.636; *p* < 0.001), and T-SAH (OR 0.807; 95% CI, 0.682–0.953; *p* < 0.012) (Figure 5).

**Table 3:** Univariate analysis of predictors for hospital mortality in all 11,068 patients.

		**Survival No. (%) (*n* = 10,135)**	**Deceased No. (%) (*n* = 933)**	***P*-value**
Sex	Male	7,788 (76.84%)	743 (79.64%)	*P* = 0.326
	Female	2,349 (23.18%)	191 (20.47%)	
Age (years)	Median	42 (IQR 25–56)	48 (IQR 33–60)	*P* < 0.001
Cause of injury	Road traffic injuries	4,344 (42.86%)	577 (61.84%)	*P* < 0.001
	Falls from heights	1,602 (15.81%)	130 (13.93%)	
	Falls from the ground	2,492 (24.59%)	133 (14.26%)	
	Assaults	975 (9.62%)	41 (4.39%)	
	Other	722 (7.12%)	52 (5.57%)	
GCS score at admission	Mild (13–15)	4,485 (44.25%)	21 (2.25%)	*P* < 0.001
	Moderate (9–12)	3,056 (30.15%)	94 (10.08%)	
	Severe (3–8)	2,594 (25.59%)	818 (87.67%)	
ISS score at admission	Mild to moderate (1–8)	2,110 (20.82%)	0 (0.00%)	*P* < 0.001
	Serious (9–15)	3,763 (37.13%)	15 (1.61%)	
	Severe (16–25)	3,303 (32.59%)	154 (16.51%)	
	Critical (25–75)	959 (9.46%)	764 (81.89%)	
Diagnosis	CC	4,915 (48.50%)	557 (59.70%)	*P* < 0.001
	T-SAH	4,368 (43.10%)	476 (51.02%)	
	A-SDH	2,860 (28.22%)	433 (46.41%)	
	SF	1,707 (16.84%)	171 (18.33%)	
	A-EDH	2,182 (21.53%)	165 (17.68%)	
	DAI	282 (2.78%)	71 (7.61%)	
Associated injuries	Chest trauma	1,204 (11.88%)	158 (16.93%)	*P* < 0.001
	Limb injuries	1,891 (18.66%)	287 (30.76%)	
Shock	Shock	684 (6.75%)	330 (35.37%)	*P* < 0.001
	Non-shock	9,451 (93.25%)	603 (64.63%)	
Treatment	Non-surgical	5,170 (51.01%)	257 (27.55%)	*P* < 0.001
	Surgical	4,965 (48.99%)	676 (72.45%)	
Length of stay (d)	ICU length (d)	2 (IQR 0–5)	1 (IQR 1–5)	*P* < 0.001
	ICU length (d)	2 (IQR 0–5)	1 (IQR 1–5)	

GCS, Glasgow Coma Scale; ISS, injury severity score; CC, cerebral contusion; T-SAH, traumatic subarachnoid hemorrhage; A-SDH, acute subdural hematoma; SF, skull fractures (including the base of skull fractures); A-EDH, acute epidural hematoma; DAI, diffuse axial cord injury.

### Nomogram

Univariate and binary logistic analyses were performed with the training cohort (*n* = 9,949, 91.30%). In the training cohort, several factors were markedly associated with poor prognosis: age, cause of injury, GCS score at admission, ISS score at admission, CC, SAH, SDH, SF, EDH, shock, associated injuries, and treatment (*p* < 0.001) (Table 4).

**Table 4:** Univariate analysis of the training cohort.

		**Good prognosis**	**Poor prognosis**	***P*-value**	**OR (95% CI)**
		**NO. (%)**	**NO. (%)**		
Total		8,086 (81.27)	1,863 (18.73)		
Sex				*P* = 0.472	
	Male	6,222 (81.1)	1,448 (18.9)		
	Female	1,864 (81.8)	415 (18.2)		
Age (years)				*P* < 0.001	
	0–14 years	1,097 (91.5)	102 (8.5)		
	15–44 years	3,627 (84)	693 (16)	*P* < 0.001	2.055 (1.651–2.557)
	45–65 years	2,586 (77.4)	757 (22.6)	*P* < 0.001	3.148 (2.53–3.917)
	Over 65 years	776 (71.4)	311 (28.6)	*P* < 0.001	4.31 (3.384–5.489)
Cause of injury				*P* < 0.001	
	Road traffic injuries	3,569 (79.3)	934 (20.7)		
	Falls from heights	1,244 (79.7)	317 (20.3)	*P* = 0.715	0.974 (0.844–1.123)
	Falls from the ground	1,918 (84.2)	359 (15.8)	*P* < 0.001	0.715 (0.626–0.818)
	Assaults	835 (86.7)	128 (13.3)	*P* < 0.001	0.586 (0.48–0.715)
	Other	520 (80.6)	125 (19.4)	*P* = 0.424	0.919 (0.746–1.131)
GCS score at admission				*P* < 0.001	
	Mild	3,772 (93.1)	281 (6.9)		
	Moderate	2,382 (83.6)	467 (16.4)	*P* < 0.001	2.632 (2.25–3.078)
	Severe	1,932 (63.4)	1,115 (36.6)	*P* < 0.001	7.747 (6.722–8.928)
ISS score at admission				*P* < 0.001	
	Mild (ISS 1–8)	1,902 (98.1)	37 (1.9)		
	Serious (ISS 9–15)	3,409 (97.3)	95 (2.7)	*P* = 0.067	1.433 (0.976–2.103)
	Severe (ISS 16–25)	2,570 (83.2)	518 (16.8)	*P* < 0.001	10.361 (7.384–14.539)
	Critical (ISS 25)	205 (14.5)	1,213 (85.5)	*P* < 0.001	304.17 (212.76–434.85)
CC	Yes	3,761 (77.5)	1,089 (22.5)	*P* < 0.001	1.618 (1.461–1.792)
	No	4,325 (84.8)	774 (15.2)		
SAH	Yes	3,226 (77)	965 (23)	*P* < 0.001	1.619 (1.463–1.791)
	No	4,860 (84.4)	898 (15.6)		
SDH	Yes	1,934 (71.3)	778 (28.7)	*P* < 0.001	2.281 (2.053–2.534)
	No	6,152 (85)	1,085 (15)		
SF	Yes	1,338 (79.1)	354 (20.9)	*P* = 0.011	1.183 (1.039–1.347)
	No	6,748 (81.7)	1,509 (18.3)		
EDH	Yes	2,122 (73)	784 (27)	*P* = 0.001	0.81 (0.712–0.922)
	No	5,964 (84.7)	1,079 (15.3)		
Shock	Yes	279 (30.8)	627 (69.2)	*P* < 0.001	14.195 (12.178–16.546)
	No	7,807 (86.3)	1,236 (13.7)		
Concomitant diagnosis	Yes	2,122 (73)	784 (27)	*P* < 0.001	2.042 (1.84–2.267)
	No	5,964 (84.7)	1,079 (15.3)		
Treatments	Non-surgical	4,612 (90.7)	472 (9.3)	*P* < 0.001	3.912 (3.493–4.382)
	Surgical	3,474 (71.4)	1,391 (28.6)		

GCS, Glasgow Coma Scale; ISS, injury severity score; CC, cerebral contusion; T-SAH, traumatic subarachnoid hemorrhage; A-SDH, acute subdural hematoma; SF, skull fractures (including the base of skull fractures); A-EDH, acute epidural hematoma; DAI, diffuse axial cord injury.

Independent variables were determined by stepwise regression analysis. After excluding the variables with poor prediction performance or multicollinearity, the following eight variables with prognostic significance were obtained: age (45–65 vs. 0–14 years) [*p* < 0.001; OR 1.39 (95% CI: 1.033–1.872)], age (over 65 vs. 0–14 years) [*p* < 0.001; OR 1.919 (95% CI: 1.367–2.696)], GCS (moderate vs. mild) [*p* < 0.001; OR 1.709 (95% CI:1.384–2.11)], GCS (severe vs. mild) [*p* < 0.001; OR 3.256 (95% CI:2.68–3.957)], ISS (serious vs. mild) [*p* < 0.04; OR 1.509 (95% CI: 1.019–2.232)], ISS (severe vs. mild) [*p* < 0.04; OR 6.578 (95% CI: 4.644–9.316)], ISS (critical vs. mild) [*p* < 0.001; OR 145.106 (95% CI: 99.855–210.864)], SDH [*p* < 0.001; OR 1.391 (95% CI: 1.18–1.64)], EDH [*p* < 0.008; OR 0.769 (95% CI:0.633–0.934)], shock [*p* < 0.001; OR 5.897 (95% CI: 4.746–4.746)], accompanying injury [*p* < 0.014; OR 0.805 (95% CI: 0.677–0.956)], and treatment [*p* < 0.001; OR 1.433 (95% CI: 1.207–1.702)] (Table 5).

**Table 5:** Multivariate logistic regression analysis on the training cohort.

		***P*-value**	**OR (95% CI)**
Age (years)		*P* < 0.001	
	0–14 years^*^	Ref	Ref
	15–44 vs. 0–14 years	*P* = 0.538	1.097 (0.818–1.471)
	45–65 vs. 0–14 years	*P* = 0.03	1.39 (1.033–1.872)
	Over 65vs. 0–14 years	*P* < 0.001	1.919 (1.367–2.696)
GCS score at admission		*P* < 0.001	
	Mild^*^	Ref	Ref
	Moderate vs. mild	*P* < 0.001	1.709 (1.384–2.11)
	Severe vs. mild	*P* < 0.001	3.256 (2.68–3.957)
ISS score at admission		*P* < 0.001	
	Mild to moderate (ISS 1–8)^*^	Ref	Ref
	Serious (ISS 9–15) vs. (ISS 1–8)	*P* = 0.04	1.509 (1.019–2.232)
	Severe (ISS 16–25) vs. (ISS 1–8)	*P* < 0.001	6.578 (4.644–9.316)
	Critical (ISS 25) vs. (ISS 1–8)	*P* < 0.001	145.11 (99.86–210.86)
SDH	Yes	*P* < 0.001	1.391 (1.18–1.64)
EDH	Yes	*P* = 0.008	0.769 (0.633–0.934)
Shock	Yes	*P* < 0.001	5.897 (4.746–7.326)
Associated injuries	Yes	*P* = 0.014	0.805 (0.677–0.956)
Treatment	Surgical	*P* < 0.001	1.433 (1.207–1.702)

GCS, Glasgow Coma Scale; ISS, injury severity score; SDH, subdural hematoma; EDH, epidural hematoma; OR, odds ratio; 95% CI 95%, confidence interval. ^*^Control group.

**Figure 6:**
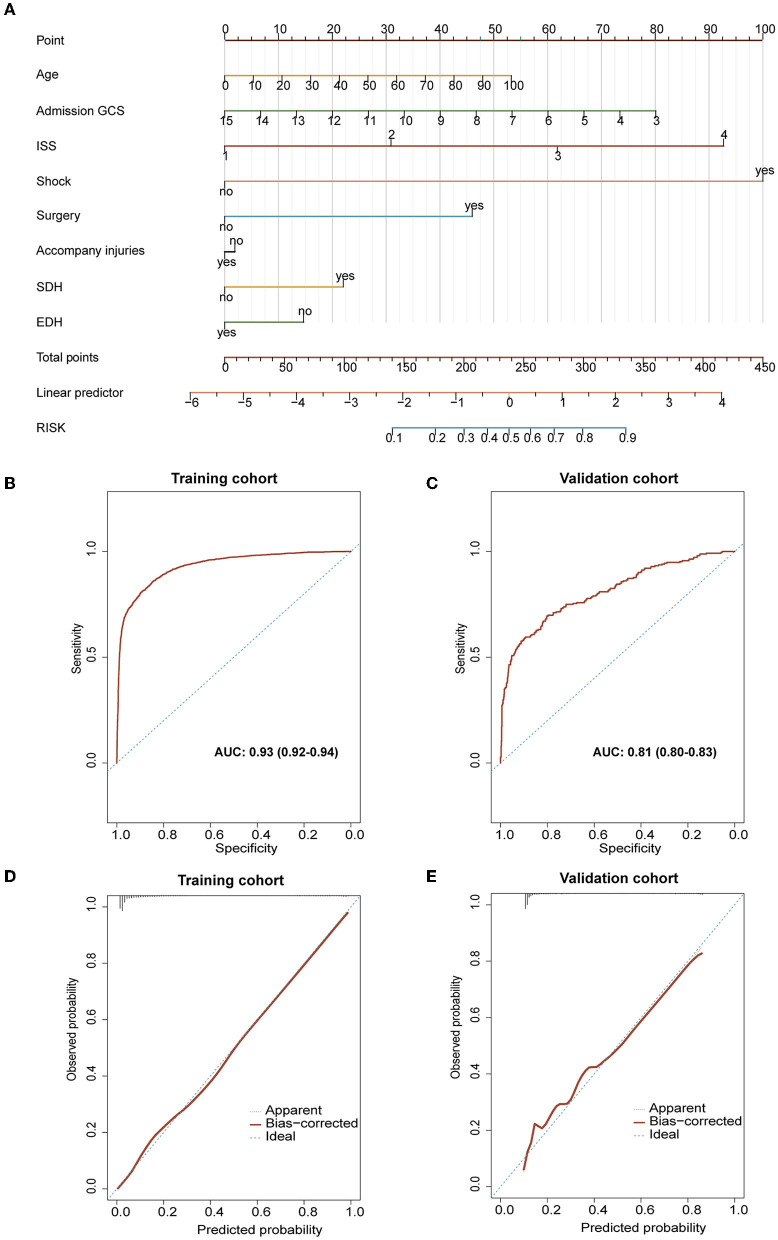
Evaluation of related characteristics as prognostic indicators for the prognosis of TBI. **(A)** The prediction model of poor prognosis was established and expressed by nomogram. **(B, C)** The results of calibration and ROC curve analysis of the purposed nomogram. **(B)** The AUC of the purposed nomogram for predicting poor prognosis in the training cohort was 93.04% (95% CI: 92.3–93.70). **(C)** The calibration curve analysis of the nomogram in the training cohort. The dashed line stands for perfect prediction. The dotted line represents apparent estimates of predicted vs. observed values, meanwhile, the solid line (on behalf of bias) shows the corrected estimates *via* employing 1,000 bootstrap samples. ROC, receiver operating characteristic curve; AUC, the area under the ROC curve. **(D, E)** The results of calibration and ROC curve analysis of the purposed nomogram for predicting poor prognosis occurrence in the external validation cohort. **(D)** The AUC of the purposed nomogram for predicting poor prognosis in the validation cohort was 81.02% (95% CI: 80.3–82.70). **(E)** The calibration curve analysis of the nomogram in the validation cohort. The dashed line stands for perfect prediction. The dotted line represents apparent estimates of predicted vs. observed values, and the solid line (on behalf of bias) shows the corrected estimates *via* employing 1,000 bootstrap samples.

The established nomogram is presented in Figure 6A. With bootstrapping, internal validation showed that the C-statistic for the risk score was 93.04% (95% CI: 92.3–93.70) (mean absolute error was 0.05) (Figure 6B). The calibration curve analysis of this predictive model exhibited good agreement (slope of 0.8432 and intercept of −0.1108). Across the reasonable threshold probabilities, our nomogram had a higher net benefit than each factor alone (Figure 6C).

External validation was conducted using a validation cohort of 1,119 patients from 2018 to 2020. The prediction model had an accuracy, while the area under the curve (AUC) of the prediction model was 81.02% (95% CI: 80.3–82.70) (Figure 6D). Besides, calibration curve analysis of the prediction model revealed a good agreement in the validation cohort (slope of 0.8358 and intercept of −0.1107; Figure 6E).

## Discussion

The absolute number of patients with TBI in China exceeds that of most countries in the world, which has resulted in serious consequences and a huge economic burden. It is a huge challenge to increase the survival rate and cure rates for patients with TBI. Elucidation of the characteristics and changes in incidences of TBI will inform the treatment of TBI in China. In our study, based on 18-year consecutive clinical data in a tertiary hospital in West China, we retrospectively summarized the general characteristics and variation trend of patients with TBI and finally established a nomogram prediction model of poor prognosis to help future treatment and decision-making.

Notably, our current results presented a “dual-feature” on the dynamic shifting of injury patterns and affected populations in patients with TBI. In our current cohort, the patients with TBI were predominantly male (aged between 25 and 56 years), and RTI was the leading cause of death. Meanwhile, the incidence of RTI and assaults decreased over time, while the incidence of TBI increased in the GLF group. These results might be attributed to extensive social involvement and high exposure risks among middle- and young-aged men, especially with the fast-growing public transportation infrastructure and socioeconomic activities in the past decades. Evidence suggested varied injury patterns across different income countries: patients with TBI in middle-income and low-income countries were generally younger, vulnerable road-traffic users, and increased motorization, inadequate traffic education, and delayed traffic safety regulation implementation were the main causes for the TBI in these countries (15–17). In terms of high-income country counterparts, who were generally motor-vehicle occupants, studies witnessed an epidemiological shift toward an elderly population affected by TBI, who were predominantly advanced in age (>50 years) and resulting in more fallen associated contusion injuries (18–21).

Considering the rapid urbanization, improved traffic regulation, and aging population in the past decades in China, it was not surprising that our results simultaneously reflected both the characteristics of middle- and low-income countries and high-income countries. First, preventive measures improved traffic safety and reduced the incidence of traffic accidents, which especially decreased the risk for younger individuals (5, 22). Second, the implementation of the amended Criminal Law, which imposed harsher punishments on driving under the influence (DUI), demonstrated unequivocal success, and the incidence of traffic accident-related TBI has decreased by over 50% since 2011 (9). Our results also confirmed this positive phenomenon. In addition, increased life expectancy and greater mobility for the elderly population were the main contributors to the rise in the absolute incidence of TBI among the elderly (23, 24). Therefore, this shifting paradigm may highlight both the pace of rapid urbanization and aging in developing countries, especially China, which will be of great help to future public health and policymaking for the approaches of prevention, management, and post-injury care for patients with TBI.

The in-hospital mortality rate in our study was 9.33%. Previously, Wu et al. (25) reported a mortality rate of 10.8%, while Gao et al. (9) reported a rate of 5%. Discrepancies in these in-hospital mortality rates among studies implied varied accessibility of medical systems, treatment strategies, and imbalanced regional development. We found a significantly high mortality rate among patients with low GCS scores, low ISS scores, and a combined shock state on admission, indicating that excess primary injury to the brain was the primary cause of death. Therefore, early diagnosis and treatment were the key to manage severe TBI. Active interventions should be made to prevent further exacerbations of TBI.

Since 2011, the inpatient mortality rate has decreased with the introduction of ICP in our institution. Chiara et al. reported that the 6-month mortality rate was low in patients on ICP [441/1,318 (34%)], compared to patients without ICP [517/1,049 (49%); *p* < 0.0001] (26). ICP monitoring could timely detect ICP changes, rapidly demonstrate the effect sizes of surgical treatments, and indicate the necessity for further interventions in the early stages (26–30). Our results reiterated that the application of ICP was a pivotal factor in improving the prognosis of patients with TBI.

Clinical diagnosis and treatment decision models for craniocerebral injuries are vital for improving clinical outcomes for critically ill patients in the emergency and ICU departments. Currently, the two most influential prognostic models for TBI are the International Collaboration for Prognostic Clinical Testing and Research in Craniocerebral Injury (IMPACT) and the Collaboration for Randomized Studies after Craniocerebral Injury (CRASH). Our nomogram, which incorporated eight factors (age, GCS, ISS, SDH, EDH, shock, accompanying injury, and treatment), demonstrated good discrimination and calibration abilities. Compared to CRASH, our prediction model had several comparable factors, while the ISS was unique and could not be ignored based on our data. The GCS and ISS scores and shock were the leading factors and were measured by the standard deviation of the nomogram. GCS was the most important predictor of total mortality in patients with TBI, and GCS ≤8 for TBI was considered severe (31).

However, for patients with multiple injuries, combined injuries, and complex injuries, prognosis or mortality usually could not be rapidly assessed by GCS alone. The ISS was potentially a good predictor of death in trauma patients (32). A Japanese study focusing on prognostic factors for TBI found that ISS and injury mechanisms were strongly associated with mortality outcomes (4). Considering the multiple factors associated with TBI, including medical and socioeconomic elements, we proposed a more comprehensive model to reflect the dynamic changes during TBI treatment. In recent years, many nomogram models for TBI have been developed for prognostic prediction based on clinicopathological factors (12, 33). For example, a multicenter observational study proposed an algorithm based on nomograms that can predict mortality in real-time during intensive care after TBI (34). The nomogram prediction model that we established integrated the factors involved in TBI prognosis with more inclusive and specific elements, reflecting temporal shifts in TBI therapeutic approaches and regimes (35).

In the analysis of mortality and prognostic factors, it is noteworthy that patients with EDH seem to have a better prognosis. Comparative analysis based on our study found that patients in the EDH group were younger than those in the non-EDH group (5 vs. 13.7%), had a lower incidence of shock at admission (6.90 vs. 9.80%), and had a higher rate of surgery (64.60 vs. 47.30%). There have been some consistent reports in previous studies. Taussky et al. (36) observed that the mortality rate of SDH was 41% (19/46), while the mortality rate of patients with EDH was 3% (1/37). Therefore, patients with EDH in TBI seem to have a better prognosis.

## Limitations

As a single-center retrospective study spanning decades, data from patients' long-term follow-up were unretrievable for certain cases. In addition, with changes in treatment modalities over 18 years, data heterogeneity was inevitable, resulting in the inability to further certain stratifications. Regarding the establishment of the prediction model, our results were relatively optimistic. However, external data for consistency and calibration evaluation were warranted.

## Conclusion

Most of the patients with TBI were middle-aged men, and RTI was the leading cause of death. Low GSC score, high ISS score, or concomitant shock state at admission were independent risk factors for TBI. The incidence of TBI was increasing in people over 45 years of age. The incidence of RTI and assaults declined over time, while the incidence of GLF increased. Finally, we proposed a nomogram for poor prognosis and validated its efficacy with good reliability, which may help in the exploration and research of future prevention and treatment of TBI. Our findings demonstrated the demographic characteristic changes and regime shifts of patients with TBI in a large city in western China and may provide experience for the future treatment of TBI, especially in developing countries with a rapid process of urbanization.

## Data availability statement

The original contributions presented in the study are included in the article/Supplementary material, further inquiries can be directed to the corresponding authors.

## Ethics statement

The studies involving human participants were reviewed and approved by the Institutional Research Board of Tangdu Hospital, Fourth Military Medical University (TDLL-202207-08). Written informed consent from the patients/participants or patients/participants' legal guardian/next of kin was not required to participate in this study in accordance with the national legislation and the institutional requirements.

## Author contributions

SGu had full access to the entire data in the study and took responsibility for the integrity and accuracy of the data analysis. Concept and design: PJ, YW, LW, and YQ. Acquisition and analysis or interpretation of data: SGu, RH, FC, PJ, JL, YZ, MC, WZ, YJ, SGe, YW, LW, and YQ. Drafting of the manuscript: SGu, RH, FC, CF, and YJ. Critical revision of the manuscript for important intellectual content: SGu, RH, FC, YW, and LW. Statistical analysis: SGu, RH, FC, PJ, JL, YZ, MC, WZ, TH, and NW. Obtained funding: YW, LW, and YQ. Administrative, technical, or material support: SGu, RH, FC, SGe, JL, YZ, MC, WZ, TH, NW, and YJ. Supervision: TH, SGe, YW, LW, and YQ. All authors reviewed the manuscript, contributed to the article, and approved the submitted version.

## Supplementary material

Supplementary Figure 1 Some of the other features of brain injury. **(A)** The most common causes of different degrees of brain damage vary. Assault (59.35%) is the most common cause of mild brain injury, and traffic accident (36.96%) is the most common cause of severe brain injury. **(B–D)** The onset time of patients with TBI showed certain regularity in the month, week, and day. **(B)** TBI occurred most in September (1,034 cases, 9.34%) and least in February (735 cases, 6.64%). **(C)** The incidence rate was the highest on Saturday and Sunday (14.76% and 14.57%). **(D)** The peak time point was 17:00 [*n* = 781 (8.1%)] every day. 05:00–06:00 is the low time point.

Supplementary Figure 2 From 2003 to 2020, the general characteristics of patients with TBI showed a certain trend over time. **(A, B)** Gender and treatment change over time. **(C, D)** TBI severity (GCS and ISS) changes over time.

Supplementary Table 1 Univariate analysis of training cohort and validation cohort.
