# Supplementary figures and images for: Epidemiological characteristics for patients with traumatic brain injury and the nomogram model for poor prognosis: an 18-year hospital-based study
Source: Front Neurol. 2023 May 23;14:1138217. doi: 10.3389/fneur.2023.1138217 (PMC10242078; doi:10.3389/fneur.2023.1138217)

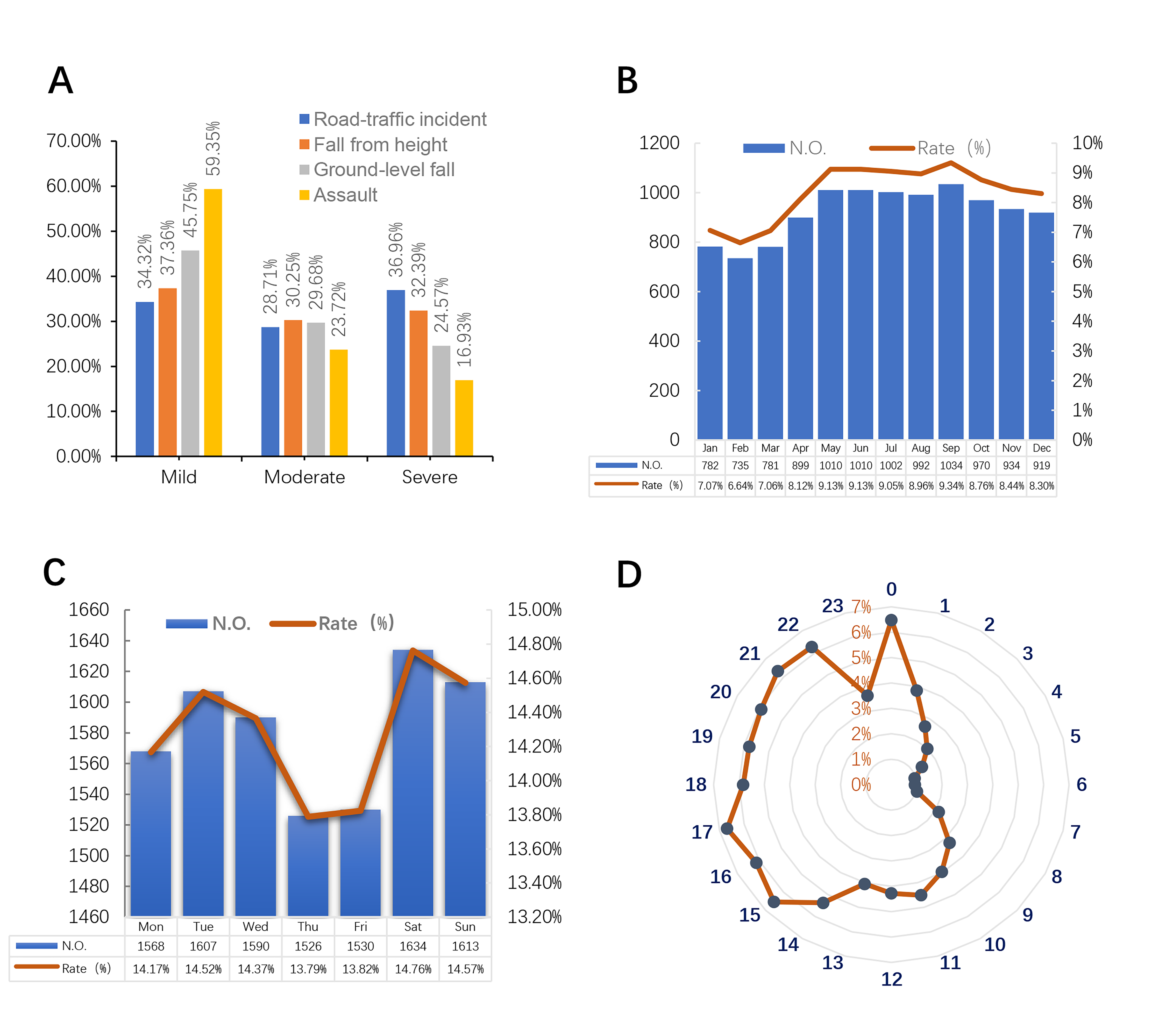

Supplement: Supplementary Figure 1 — Some of the other features of brain injury. (A) The most common causes of different degrees of brain damage vary. Assault (59.35%) is the most common cause of mild brain injury, and traffic accident (36.96%) is the most common cause of severe brain injury. (B–D) The onset time of patients with TBI showed certain regularity in the month, week, and day. (B) TBI occurred most in September (1,034 cases, 9.34%) and least in February (735 cases, 6.64%). (C) The incidence rate was the highest on Saturday and Sunday (14.76% and 14.57%). (D) The peak time point was 17:00 [n = 781 (8.1%)] every day. 05:00–06:00 is the low time point. [file Image_1.TIF]

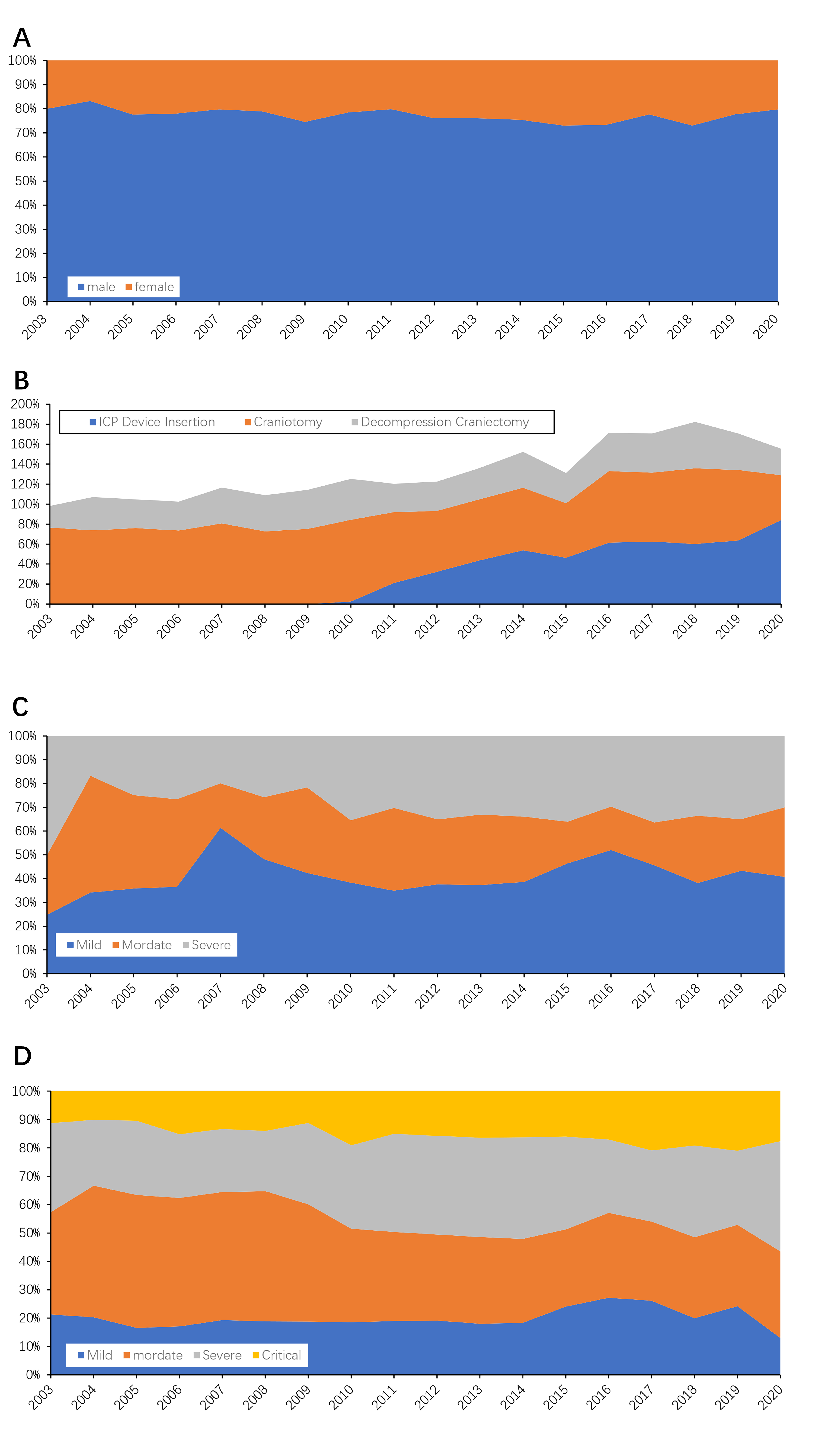

Supplement: Supplementary Figure 2 — From 2003 to 2020, the general characteristics of patients with TBI showed a certain trend over time. (A, B) Gender and treatment change over time. (C, D) TBI severity (GCS and ISS) changes over time. [file Image_2.TIF]
