# Supplementary material for: Epidemiological characteristics for patients with traumatic brain injury and the nomogram model for poor prognosis: an 18-year hospital-based study
Source: Front Neurol. 2023 May 23;14:1138217. doi: 10.3389/fneur.2023.1138217 (PMC10242078; doi:10.3389/fneur.2023.1138217)
Supplement: Supplementary Table 1 — Univariate analysis of training cohort and validation cohort. [file Table_1.DOCX]

SUPPLEMENT Table1 Univariate analysis of training cohort and validation cohort

|  |  | Training cohort | Validation cohort | P value |
| --- | --- | --- | --- | --- |
|  |  | NO. (%) | NO. (%) |  |
| Total |  | 9,949(89.89) | 1,119(10.11) |  |
| Sex | Male | 7,670(77.09) | 859(76.76) | P=0.834 |
|  | Female | 2,279(22.91) | 260(23.24) |  |
| Age (years) | Median | 42(IQR 24-55) | 52(IQR 37-63) | P<0.001 |
|  | 0-14years | 1,199(12.05) | 103(9.2) |  |
|  | 15-44years | 4,320(43.42) | 275(24.58) |  |
|  | 45-65years | 3,343(33.6) | 517(46.2) |  |
|  | Over 65years | 1,087(10.93) | 224(20.02) |  |
| Cause of injury | Road traffic injuries | 4,503(45.26) | 418(37.35) | P<0.001 |
|  | Falls from heights | 1,561(15.69) | 171(15.28) |  |
|  | Falls from the ground | 2,277(22.89) | 348(31.1) |  |
|  | Assaults | 963(9.68) | 53(4.74) |  |
|  | Other | 645(6.48) | 129(11.53) |  |
| GCS score at admission | Median | 12(IQR 8-14) | 11(IQR 7-13) | P=1.000 |
|  | Mild | 4,053(40.74) | 453(40.48) |  |
|  | Moderate | 2,849(28.64) | 301(26.9) |  |
|  | Severe | 3,047(30.63) | 365(32.62) |  |
| ISS score at admission | Mild (ISS 1-8) | 1,939(19.49) | 216(19.3) | P=0.292 |
|  | Serious (ISS9-15） | 3,504(35.22) | 522(46.65) |  |
|  | Severe (ISS16-25） | 3,088(31.04) | 232(20.73) |  |
|  | Critical (ISS 25） | 1,418(14.25) | 149(13.32) |  |
| CC | Yes | 4,850(48.75) | 622(55.59) | P=0.396 |
|  | No | 5,099(51.25) | 497(44.41) |  |
| SAH | Yes | 4,191(42.12) | 653(58.36) | P=0.034 |
|  | No | 5,758(57.88) | 466(41.64) |  |
| SDH | Yes | 2,712(27.26) | 581(51.92) | P<0.001 |
|  | No | 7,237(72.74) | 538(48.08) |  |
| SF | Yes | 1,692(17.01) | 186(16.62) | P=1.000 |
|  | No | 8,257(82.99) | 933(83.38) |  |
| EDH | Yes | 2,075(20.86) | 272(24.31) | P=0.735 |
|  | No | 7,874(79.14) | 847(75.69) |  |
| Shock | Yes | 906(9.11) | 108(9.65) | P=1.000 |
|  | No | 9,043(90.89) | 1,011(90.35) |  |
| Concomitant diagnosis | Yes | 2,906(29.21) | 427(38.16) | P=0.231 |
|  | No | 7,043(70.79) | 692(61.84) |  |
| Treatment | Non-surgical | 5,084(51.1) | 343(30.65) | P=0.006 |
|  | Surgical | 4,865(48.9) | 776(69.35) |  |
| Prognosis | Good prognosis | 8,086(81.27) | 867(77.48) | P=0.602 |
|  | Poor prognosis | 1,863(18.73) | 252(22.52) |  |

GCS: Glasgow Coma Scale, ISS: Injury Severity Score

CC: cerebral contusion, T-SAH: traumatic subarachnoid hemorrhage, A-SDH: acute subdural hematoma, SF: skull fractures (including base of skull fractures), A-EDH: acute epidural hematoma, DAI: diffuse axial cord injury
